# The Association Between Persistence and Adherence to Disease-Modifying Therapies and Healthcare Resource Utilization and Costs in Patients With Multiple Sclerosis

**DOI:** 10.36469/jheor.2022.33288

**Published:** 2022-04-26

**Authors:** Gabriel Pardo, Elmor D. Pineda, Carmen D. Ng, Daniel Sheinson, Nicole G. Bonine

**Affiliations:** 1 Oklahoma Medical Research Foundation; 2 Genentech, Inc.

**Keywords:** multiple sclerosis, disease-modifying therapies, persistence, adherence, healthcare resource utilization

## Abstract

**Background:** Persistence and adherence to disease-modifying therapies (DMTs) affects treatment efficacy and economic outcomes, both of which contribute to overall patient disease burden. Current literature suggests that patients with multiple sclerosis (MS) who adhere to DMT for 12 months have fewer relapses and reduced MS-related healthcare resource utilization (HCRU) and medical costs than nonadherent patients.

**Objective:** To expand on previous research by estimating the association of persistence and adherence with all-cause and MS-related HCRU and non-DMT costs of patients with MS across 12 and 24 months of therapy use.

**Methods:** This study was a retrospective analysis of adult patients with MS in the IBM MarketScan Commercial and Medicare Supplemental databases using claims data between April 2016 and December 2019. The index date was defined as the initiation of the DMT. Patients were required to have ≥12 months’ continuous enrollment pre-index and ≥12 or ≥24 months’ continuous enrollment post-index. Persistence was defined as no gap in DMT supply for ≥60 days within the post-index period or switch to another DMT. Adherence was calculated using the proportion of days covered (for this study, number of days covered by the DMT was 365 or 730 days), with ≥80% proportion of days covered considered adherent. Multivariable analyses were conducted to estimate total and individual components of non-DMT costs by persistence and adherence while controlling for baseline differences.

**Results:** Patients who were persistent with medication for 12 months showed a reduction in mean total non-DMT medical costs of $10 022 compared with nonpersistent patients; these savings nearly doubled ($19 230) after 24 months of persistence. A similar pattern was observed for adherent vs nonadherent patients (reduction in costs at 12 months, $8543; at 24 months, $16 091). The largest reduction in all-cause HCRU costs was observed in the inpatient setting, while the largest reduction in MS-related costs was observed in the outpatient setting.

**Discussion:** Patients with MS who were persistent and adherent to medication had substantially lower all-cause and MS-related non-DMT medical costs compared with those who were nonpersistent or nonadherent.

**Conclusions:** These findings further support the importance of persistence and adherence to DMTs in patients with MS.

## BACKGROUND

Multiple sclerosis (MS) is a chronic, progressive inflammatory disease characterized by relapses that can lead to neurological disability.^1^ MS affects nearly 1 million people living in the United States.^2^ Following a prescribed medication regimen is critical for patients with MS to derive the benefits of treatment and achieve their therapeutic goals (eg, inflammatory disease activity control and no evidence of disease progression). However, deviations from prescribed treatment regimens often occur (eg, missed doses or self-discontinued therapy), and these lapses can have a significant effect on drug efficacy, leading to poor disease control, increased healthcare resource utilization (HCRU), and greater medical costs.^3^

Persistence and adherence to medication translates into reduced disease burden for patients and are associated with more favorable economic outcomes. Using predicted mean costs for the post-index year, Burks et al^3^ estimated that disease-modifying therapy (DMT) adherence could reduce total non-DMT medical costs by 41.7%, hospitalization costs by 58.5%, emergency room costs by 46.9%, and outpatient admission costs by 32.9% (amounting to a decrease in total annual medical expenses of $5816 per patient). Adherence to DMTs in patients with MS was associated with a 42% reduction in relapses, a 38% reduction in hospitalizations, and a 52% reduction in emergency room visits.^3^ Another study reported that nonadherent patients had a mean increase of 89% in medical costs over those who were adherent.^4^ In addition to physical and emotional burdens, medical costs comprise another component of the overall disease burden for patients with MS. Increased medical costs can cause psychological stress and can affect other aspects of life, due to dwindling financial reserves that are spent on disease management. In addition to patient economic burdens, increased HCRU costs apply pressure to the economy, with estimated yearly increases in MS management costs of $8 million.^5^

A recent analysis of a real-world cohort of more than 12 000 patients with MS showed that nearly 40% of patients were not adherent to their prescribed DMT.^3^ This rate of nonadherence is deeply concerning and merits further investigation into the consequences of deviating from MS DMT regimens, which patients may be most at risk, and which components of HCRU may be driving these costs. Using the same database as Burks et al,^3^ this study evaluated the effects of persistence and adherence on all-cause and MS-related non-DMT costs over 12 months and 24 months of DMT use.

## METHODS

### Data Source

This study was a retrospective claims analysis using IBM MarketScan® Commercial and Medicare Supplemental Claims databases^6^ covering a sample of commercially insured patients in the United States. The MarketScan® Database contains deidentified data on medical and pharmacy claims for 263 million members, from over 160 large US employers and 40 contributing health plans. The commercial database, which is the largest component of MarketScan®, comprises adults under 65 years of age and their dependents; the Medicare database is composed of retired individuals who are covered by previous employers. This analysis utilized inpatient and outpatient claims, diagnoses, and procedures based on the *International Classification of Diseases, Ninth/Tenth Revision, Clinical Modification*; Current Procedural Terminology (CPT) codes; and Healthcare Common Procedure Coding System (HCPCS) from these databases.

### Patient Selection and Identification

Patients at least 18 years of age with a diagnosis of MS from 2016 onward who initiated ocrelizumab, fingolimod, dimethyl fumarate, glatiramer acetate, interferon beta-1a/b, natalizumab, or teriflunomide between April 2017 and December 2019 were identified. The date of initiation of the new DMT was considered the index date. Patients were required to have at least 12 months of continuous enrollment pre-index and at least 12 or 24 months post-index (necessitating initiation of the DMT by December 2017 or December 2018, depending on the length of follow-up). Patients were required to have at least 2 prescriptions or infusions of index DMTs. For ocrelizumab, evidence of the first dose (2 infusions between 13 and 21 days apart, with no subsequent dose for at least 100 days) was required. Patients receiving ocrelizumab were identified by HCPCS J/C codes (J2350, C9494), National Drug Code (502-42015-001), or all of the following criteria: (1) miscellaneous HCPCS J codes (J3490, J3590, J9999, C9399) on or after April 1, 2017, which included CPT codes (96413, 96415, 96365, 96366) indicating intravenous infusion procedures within ±1 day; (2) an MS diagnosis on the same day as any miscellaneous HCPCS J code *or* any MS DMT use in the year prior to the earliest identified miscellaneous HCPCS J code; (3) no MS DMT use (other than ocrelizumab) up to 6 months after the earliest identified miscellaneous HCPCS J code. These criteria were based on a published algorithm to identify patients receiving ocrelizumab before J and C codes were assigned.^7^ Patients receiving any off-label therapies (eg, rituximab) or those who initiated multiple DMTs on the index date were excluded. Patients receiving alemtuzumab were excluded because treatment is limited to 2 treatment courses, and patients initiating mitoxantrone were excluded due to a limited sample size.

### Study Measures

Patient demographics, including age, sex, region, payer, and plan type, were collected at the index date. Clinical characteristics and DMT treatment patterns were based on medical and pharmacy claims in the 12 months prior to the index date. These covariates included history of relapse, prior DMT use, Charlson Comorbidity Index (CCI), and presence of various MS symptoms (**Supplementary Table S1**). Relapses were identified in the pre-index year by any of the following: any inpatient visit with a principal MS diagnosis; any outpatient visit with an MS diagnosis and evidence of a prescription for a high-dose oral steroid (500 mg/day prednisone equivalent or higher); and injectable steroid or plasma exchange/intravenous immunoglobulin administration within 30 days of the outpatient visit.^8,9^

Persistence was defined as having no gap in DMT supply for at least 60 days within 12 or 24 months of the post-index date. Additionally, patients who had evidence of any other DMT use besides the index DMT within 12 or 24 months of the post-index date were also considered not persistent. Days of supply for fills of oral and injectable DMTs were determined directly from claims, while days of supply for ocrelizumab and natalizumab were defined as 182 days and 28 days, respectively, from the start of each dose (ie, first infusion of the loading dose). Adherence was calculated using the proportion of days covered (PDC) measure, which calculates the number of days covered by the medication out of a fixed length of time. For this study, PDC was calculated as the proportion of days covered by supply of the index DMT out of 12 or 24 months post-index, depending on the analysis (365 and 730 days). Each day during the year was considered covered or not covered by supply of DMT depending on the number of days supplied from the previous fill or administration. All days during the year after evidence of a switch to a different DMT were considered uncovered by supply. Overlapping days were not carried forward if the patient received an infusion treatment early; however, overlapping days were carried forward in the injectable and oral groups if patients received their prescription early. A PDC of 80% or higher was considered adherent.

HCRU and non-DMT costs were calculated for all-cause and MS-related costs over 12 and 24 months of continuous post-index enrollment. The following non-DMT costs were analyzed: costs associated with outpatient visits, inpatient visits, and non-DMT pharmacy fills.

### Statistical Analysis

Patient demographic and disease characteristics were tabulated by persistent vs nonpersistent status and separately for adherent vs nonadherent status. Generalized linear models with a γ distribution and log link function were used to estimate all-cause and MS-related non-DMT costs for persistent and nonpersistent patients and for adherent and nonadherent patients. Cost estimates were adjusted for age, sex, region, payer type (commercial vs Medicare), insurance plan type, relapse in the prior year (yes/no), DMT use in the pre-index year (yes/no), CCI, presence of MS symptoms (yes/no), and pre-index non-DMT costs. Pre-index costs were included in the models to control for baseline differences in HCRU.

## RESULTS

After patient attrition, a total of 4396 patients were continuously enrolled for at least 12 months after the index date, and a total of 1710 patients were enrolled for at least 24 months after the index date (**Supplementary Figure S1**). The number of patients who were persistent or adherent at 12 and 24 months is also displayed. Patient demographics and clinical characteristics were similar at 12 and 24 months. At 24 months, persistent and adherent patients were more likely to be between the ages of 45 and 54 years and to have had pre-index DMT use (*P*=.001) (Table 1). The proportion of patients initiating each DMT type also significantly varied across persistent/adherent and nonpersistent/ nonadherent groups (*P*=.001), with a plurality of persistent/adherent patients consisting of patients initiating ocrelizumab.

**Table 1.** Patient Demographic and Clinical Characteristics<sup>a</sup>

	**Persistent (n=959)**	**Nonpersistent (n=751)**	***P* Value**	**Adherent (n=996)**	**Nonadherent (n=714)**	***P* Value**
Age at index, years; mean (SD)	47.4 (10.0)	45.5 (11.8)	.00246	47.3 (10.2)	45.5 (11.8)	.00187
Age categories, n (%)						
<35 years	109 (11.4)	133 (17.7)	<.001	116 (11.6)	126 (17.6)	<.001
35-44 years	249 (26.0)	203 (27.0)		255 (25.6)	197 (27.6)	
45-54 years	351 (36.6)	222 (29.6)		365 (36.6)	208 (29.1)	
≥55+ years	250 (26.1)	193 (25.7)		260 (26.1)	183 (25.6)	
Female, n (%)	698 (72.8)	580 (77.2)	.041	718 (72.1)	560 (78.4)	.00349
Region, n (%)						
North Central	212 (22.1)	148 (19.7)	.442	218 (21.9)	142 (19.9)	.611
Northeast	216 (22.5)	164 (21.8)		221 (22.2)	159 (22.3)	
South	429 (44.7)	352 (46.9)		450 (45.2)	331 (46.4)	
West	100 (10.4)	87 (11.6)		105 (10.5)	82 (11.5)	
Unknown	2 (0.2)	0 (0)		2 (0.2)	0 (0)	
Payer, n (%)						
Commercial	932 (97.2)	718 (95.6)	.103	967 (97.1)	683 (95.7)	.147
Medicare	27 (2.8)	33 (4.4)		29 (2.9)	31 (4.3)	
Plan type, n (%)						
Comprehensive	33 (3.4)	48 (6.4)	.0504	38 (3.8)	43 (6.0)	.109
EPO/HMO	101 (10.5)	77 (10.3)		100 (10.0)	78 (10.9)	
POS	34 (3.5)	39 (5.2)		38 (3.8)	35 (4.9)	
PPO	561 (58.5)	416 (55.4)		580 (58.2)	397 (55.6)	
CDHP	155 (16.2)	107 (14.2)		162 (16.3)	100 (14.0)	
HDHP	65 (6.8)	54 (7.2)		70 (7.0)	49 (6.9)	
Unknown/missing	10 (1.0)	10 (1.3)		8 (0.8)	12 (1.7)	
Pre-index relapse, n (%)	310 (32.3)	259 (34.5)	.373	330 (33.1)	239 (33.5)	.924
Pre-index DMT use, n (%)	573 (59.7)	312 (41.5)	<.001	589 (59.1)	296 (41.5)	<.001
CCI, n (%)						
0	730 (76.1)	566 (75.4)	.888	761 (76.4)	535 (74.9)	.776
1	104 (10.8)	87 (11.6)		109 (10.9)	82 (11.5)	
2+	125 (13.0)	98 (13.0)		126 (12.7)	97 (13.6)	
MS symptom, n (%)						
Other causes of myelitis	2 (0.2)	1 (0.1)	1	2 (0.2)	1 (0.1)	1
Demyelinating disease of central nervous system	149 (15.5)	126 (16.8)	.531	156 (15.7)	119 (16.7)	.624
Disorders of optic nerve and visual pathways	125 (13.0)	88 (11.7)	.457	128 (12.9)	85 (11.9)	.61
Neurogenic bladder NOS	93 (9.7)	53 (7.1)	.064	96 (9.6)	50 (7.0)	.0664
Other disorders of soft tissues: neuralgia, neuritis, and radiculitis, unspecified	38 (4.0)	29 (3.9)	1	39 (3.9)	28 (3.9)	1
General symptoms: dizziness and giddiness	106 (11.1)	98 (13.0)	.235	114 (11.4)	90 (12.6)	.513
General symptoms: fatigue and malaise	336 (35.0)	235 (31.3)	.115	349 (35.0)	222 (31.1)	.0979
Total pre-index non-DMT costs, mean $US (SD)	21 800 (29 700)	27 900 (80 600)	.297	21 800 (29 700)	28 300 (82 300)	.279
Index DMT						
Dimethyl fumarate	150 (15.6)	147 (19.6)	<.001	154 (15.5)	143 (20.0)	<.001
Fingolimod	116 (12.1)	66 (8.8)		124 (12.4)	58 (8.1)	
Glatiramer acetate	94 (9.8)	179 (23.8)		99 (9.9)	174 (24.4)	
Interferon beta-1a	23 (2.4)	55 (7.3)		26 (2.6)	52 (7.3)	
Interferon beta-1b	4 (0.4)	10 (1.3)		4 (0.4)	10 (1.4)	
Natalizumab	66 (6.9)	54 (7.2)		65 (6.5)	55 (7.7)	
Ocrelizumab	395 (41.2)	129 (17.2)		417 (41.9)	107 (15.0)	
Teriflunomide	111 (11.6)	111 (14.8)		107 (10.7)	115 (16.1)	

Abbreviations: CCI, Charlson Comorbidity Index; CDHP, consumer-directed health plan; DMT, disease-modifying therapy; EPO, exclusive provider organization; HDHP, high-deductible health plan; HMO, health maintenance organization; NOS, not otherwise specified; OCR, ocrelizumab; POS, point of service; PPO, preferred provider organization. ^a^Separate analyses were conducted for persistent vs nonpersistent patients and for adherent vs nonadherent patients.

**Figure 1.**
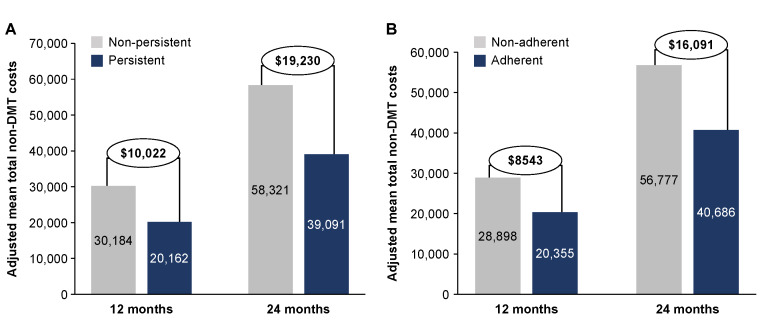
Adjusted All-Cause Non-DMT Costs at 12 and 24 Months in (**A**) Nonpersistent vs Persistent Patients or (**B**) Nonadherent vs Adherent Patients Abbreviation: DMT, disease-modifying treatment.

**Figure 2.**
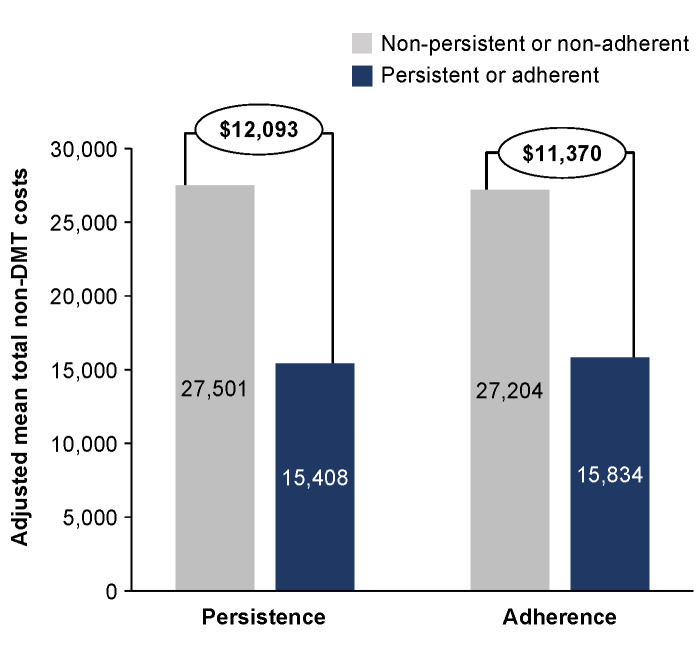
Adjusted MS-Related Non-DMT Costs at 24 Months in Nonpersistent vs Persistent Patients and Nonadherent vs Adherent Patients Abbreviations: DMT, disease-modifying therapy; MS, multiple sclerosis.

Overall, patients who were persistent with their medication for 12 months had an adjusted mean total non-DMT cost that was $10 022 lower than nonpersistent patients, and these savings nearly doubled ($19 230) after 24 months of persistence (Figure 1, A and B). A similar pattern was observed for adherent patients, who had reductions in adjusted mean total non-DMT costs of $8543 and $16 091 at 12 and 24 months, respectively, compared with nonadherent patients. For MS-related non-DMT costs at 24 months, persistent and adherent patients had adjusted mean total costs that were $12 093 and $11 370 lower, respectively, than patients who were nonpersistent or nonadherent (Figure 2). We used the unadjusted numbers to understand the distribution of specific types of costs and found that the largest reduction in all-cause non-DMT costs among persistent patients was from inpatient services (12 months, $4413; 24 months, $10 277), followed by outpatient services (12 months, $3777; 24 months, $7701) (**Supplementary Figure S2A**). The largest reduction in unadjusted MS-related non-DMT costs among persistent patients were from outpatient (12 months, $2279; 24 months, $3745) and inpatient services (12 months, $1840; 24 months, $2928) (**Supplementary Figure S2B**). Reduction of all-cause and MS-related non-DMT costs among adherent patients followed the same pattern as persistent patients, with the largest reductions in unadjusted all-cause costs coming from inpatient (12 months, $4058; 24 months, $9499) and outpatient (12 months, $2478; 24 months, $6559) services (**Supplementary Figure S3A**), and the largest reductions in unadjusted MS-related non-DMT costs from outpatient (12 months, $1208; 24 months, $3732) and inpatient (12 months, $1600; 24 months, $2812) services (**Supplementary Figure S3B**).

## DISCUSSION

Persistence and adherence to DMTs in patients with MS has been shown to affect clinical outcomes such as disease progression and relapse frequency, but it can affect economic outcomes as well, adding to patients’ overall disease burden. In this real-world analysis of a US claims database, patients with MS who were persistent and adherent with their prescribed DMT had significant reductions in non-DMT medical costs compared with those who were not persistent or adherent. The largest reduction in all-cause non-DMT costs was observed in the inpatient setting, while the largest reduction in MS-related costs was observed in the outpatient setting. A majority of patients who were persistent or adherent to their prescribed DMT were those initiating ocrelizumab. This study highlights the importance of educating patients with MS on their medications to improve adherence and persistence, which translates into cost benefits.

Overall, persistence and adherence to DMTs resulted in mean total all-cause non-DMT cost savings of nearly $20 000 and a mean total reduction in MS-related non-DMT costs of $12 000, in a 24-month period. These reductions are substantial when applying them to individual patients’ experiences and imply that in addition to achieving disease control and reducing HCRU, the financial burden of patients with MS is notably lessened by persistence and adherence. A previous analysis of this same claims database reported that nonadherent patients with MS have an increase in mean total MS-related costs of more than $5000 in a 12-month period.^3^ The present analysis reports more than double this amount ($11 370) after 24 months of nonadherence.

Understanding where HCRU costs originate can provide insight into the consequences of nonpersistence and nonadherence. HCRU costs that were from any cause showed the greatest increase in the inpatient setting, amounting to a mean increase of more than $6500 in a 24-month period. When HCRU costs that were only MS-related were evaluated, however, the greatest increase was seen in the outpatient setting, with approximately $3700 of additional associated costs being spent on average for nonpersistent or nonadherent patients in a 24-month period.

### Limitations

The potential for selection bias in patients with 12-month and 24-month follow up may have affected outcomes. Persistence and adherence were calculated based on the administration schedule recommended in each product’s prescribing information; therefore, persistence and adherence may be misclassified in instances where prescribing patterns differ from the US Food and Drug Administration–approved administration schedules. Another consideration is that other, non-MS-related medical issues could contribute to both greater HCRU costs and lower persistence and adherence. The goal of this study was to understand the relationship between persistence/adherence and costs, irrespective of which DMT was used; however, different DMTs are associated with distinct safety and tolerability profiles, which could affect costs. Although comorbidities were controlled for in this study, it is possible that new medical issues may have emerged during the follow-up period. To apply this analysis more broadly from a global perspective, these studies would need to be replicated in countries outside the United States to see if the cost savings translate. Physician practices and the healthcare landscape will differ, requiring additional research.

## CONCLUSIONS

Greater persistence and adherence to DMTs in patients with MS is associated with reduced HCRU costs for both all-cause and MS-related non-DMT costs over 24 months. These findings bolster the understanding of the economic impact that deviations from prescribed DMT use can have in patients with MS.

### Disclosures

GP has received personal compensation for serving as a consultant for Biogen, Genentech Inc, Genzyme, Greenwich Neuroscience, Celgene, EMD Serono, Horizon Therapeutics, TG Therapeutics, and Novartis. He has received personal compensation for serving on a speakers’ bureau for Biogen, BMS, Celgene, Novartis, EMD Serono, and Viela Bio. EDP is an employee of Genentech Inc and a shareholder of F. Hoffmann-La Roche Ltd. CDN is an employee of Genentech Inc and a shareholder of F. Hoffmann-La Roche Ltd. DS is an employee of Genentech Inc and a shareholder of F. Hoffmann-La Roche Ltd. NGB is an employee of Genentech Inc and a shareholder of F. Hoffmann-La Roche Ltd.

### Data Sharing Statement

The IBM MarketScan® Commercial and Medical Supplemental claims data sets analyzed during this study are not publicly available as this is proprietary information.

## Figures and Tables

**Figure:** Online Supplementary Material
